# Effects of enzyme feeding strategy on ethanol yield in fed-batch simultaneous saccharification and fermentation of spruce at high dry matter

**DOI:** 10.1186/1754-6834-3-14

**Published:** 2010-06-25

**Authors:** Kerstin Hoyer, Mats Galbe, Guido Zacchi

**Affiliations:** 1 Department of Chemical Engineering, Lund University, PO Box 124, SE-221 00 Lund, Sweden

## Abstract

**Background:**

To make lignocellulosic fuel ethanol economically competitive with fossil fuels, it is necessary to reduce the production cost. One way to achieve this is by increasing the substrate concentration in the production process, and thus reduce the energy demand in the final distillation of the fermentation broth. However, increased substrate concentration in simultaneous saccharification and fermentation (SSF) processes has been shown to result in reduced ethanol yields and severe stirring problems. Because the SSF medium is being continuously hydrolyzed, running the process in fed-batch mode could potentially reduce the stirring problems and lead to increased ethanol yields in high-solids SSF. Different enzyme feeding strategies, with the enzymes either present in the reactor from start-up or fed into the reactor together with the substrate, have been studied, along with the influence of the enzyme feeding strategy on the final ethanol yield and productivity.

**Results:**

In the present study, SSF was run successfully with 10% and 14% water-insoluble solids (WIS) in batch and fed-batch mode. The mixing of the material in the reactor was significantly better in fed-batch than batch mode, and similarly high or higher ethanol yields were achieved in fed-batch mode compared with batch SSF in some cases. No general trend in the dependence of ethanol yield on enzyme feeding strategy was found.

**Conclusions:**

The optimum enzyme feeding strategy appears to depend on the conditions during SSF, such as the WIS concentration and the concentration of inhibitory compounds in the SSF medium.

## Background

Climate change is one of the greatest challenges of our time. Replacing fossil fuels with so-called biofuels, such as bioethanol, is one way of reducing greenhouse gas emissions from the transport sector, which is responsible for a considerable proportion of total CO_2 _emissions [1]. Currently, many crops rich in sugar or starch, such as sugarcane, maize and wheat, are used for ethanol production. However, to minimize the environmental effects and the competition between crops for food and fuel production, and to maximize cost efficiency, it is important to consider other raw materials. So-called 'second-generation bioethanol production', using lignocellulosic material such as agricultural or wood residues, is considered a promising approach. In Sweden, the most abundant raw material for ethanol production is softwood, in the form of logging waste and waste from the forest industry [2].

Bioethanol can be produced from lignocellulosic material by hydrolysis of the cellulose and hemicellulose to monomeric sugars, followed by fermentation of these sugars to ethanol [1,3]. Performing hydrolysis and fermentation in a single step, the so-called 'simultaneous saccharification and fermentation' (SSF) process, has several advantages over separate saccharification and fermentation (SHF) [4-6]. In SSF, end-product inhibition of β-glucosidase is avoided, and the number of reactors needed in the process is reduced [4,7,8]. Furthermore, SSF has been shown to be superior to SHF in terms of overall ethanol yield [9-11].

**Figure 1:**
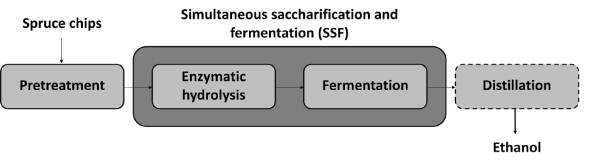
**Simplified process configuration**.

Before beginning SSF, the raw material needs to be pretreated to break down the hemicellulose and make the cellulose more accessible to the enzymes used in the hydrolysis (Figure 1). Steam explosion using SO_2 _as a catalyst has been shown to be successful for softwood and other lignocellulosic materials [2,12-18]. This results in a solid fraction containing mostly cellulose and lignin, and a hydrolysate containing monomeric sugars derived from the hemicellulose, with small amounts of other carbohydrates, sugar and lignin degradation products, acetic acid, and other compounds [19]. Some of these compounds have been found to be inhibitory to enzymatic hydrolysis [14,20-22] and fermentation [19,21,23-26].

To make ethanol an economically competitive alternative to fossil fuel, it is necessary to reduce the production cost. Recovery of ethanol from the fermentation broth by distillation is one of the most energy-intensive steps in the wood to ethanol conversion process [7,8]. Major cost savings could thus be achieved by reducing the energy demand in the distillation step. Because the cost of distillation decreases as the ethanol concentration in the distillation feed increases [8], it is important to reach the highest possible ethanol concentration in the SSF step [7]. One way of achieving this is by increasing the substrate concentration in SSF. Previous studies have shown high ethanol yields in SSF using 5% water-insoluble solids (WIS) [9,27,28]; however, studies with higher dry matter (DM) content have shown a decrease in ethanol yield [9,27-29].

One reason for the decrease in ethanol yield at higher DM content is difficulty in stirring [29], which can be overcome by running SSF in fed-batch mode, as the fibers will then be continuously degraded, reducing the viscosity of the fermentation medium compared with batch SSF. Another advantage of fed-batch SSF over batch SSF is the lower concentrations of inhibitory compounds, as all the hydrolysate is not added at the same time [30]. This also gives the yeast a chance to convert some of the inhibitory compounds into compounds with lower inhibition, and thus 'detoxify' the fermentation medium [22,31,32]. Furthermore, for feedstocks rich in pentose sugars, it has been shown that higher conversion of both pentose and hexose sugars to ethanol can be achieved when keeping the glucose level low in the fermentation medium [33], which can be achieved in fed-batch SSF [33,34].

To our knowledge, the effects on overall ethanol yield and enzyme consumption of different methods of adding the enzymes to fed-batch SSF have not been studied. The aim of this study was therefore to investigate the effects of different enzyme feeding strategies to optimize the SSF process for ethanol production.

## Methods

### Raw material

Spruce was kindly provided by a sawmill in Southern Sweden (Widtsköfle Sågverk AB, Degeberga, Sweden). The wood was chipped at a knife mill (Retsch GmbH, Haan, Germany) and sieved to obtain a chip size of 2-10 mm. The chips were stored in a plastic bag at 4°C before use. The same batch of raw material was used for all experiments.

### Steam pretreatment

The softwood chips were impregnated with SO_2 _(2% w/w moisture) for 20 min at room temperature, in tightly sealed plastic bags. The amount of SO_2 _absorbed was determined by weighing the plastic bags and their contents before and after impregnation.

The impregnated softwood was pretreated in a steam pretreatment unit equipped with a 10 L reactor, as previously described by Palmqvist *et al. *[35]. All steam pretreatment experiments were performed at 210°C for 5 min, as these had previously been determined to be the optimal pretreatment conditions for high yield of fermentable sugars in enzymatic hydrolysis and high yield of ethanol in subsequent fermentation [36]. When the desired pretreatment time had elapsed, the pressure was released and the material collected in a tank. Owing to the limited size of the reactor, the impregnated softwood was pretreated in batches of 700 g DM. The slurries obtained were mixed to form one large batch. Two different batches were pretreated on different occasions (hereafter referred to as batch 1 and batch 2). The pretreated slurry was stored at 4°C before subsequent analysis and use in SSF.

### Cell cultivation

#### Inoculum

The inoculum culture was prepared on an agar plate containing pure baker's yeast (*Saccharomyces cerevisiae*), purified from compressed baker's yeast (Jästbolaget, Rotebo, Sweden). The cells were added to a 300 mL Erlenmeyer flask together with 70 mL of an aqueous solution containing 23.8 g/L glucose, 10.8 g/L (NH_4_)_2_SO_4_, 5.0 g/L KH_2_PO_4 _and 1.1 g/L MgSO_4 _7H_2_O. The solution also contained 14.4 g/L trace metal solution and 1.4 g/L vitamin solution, prepared as described by Taherzadeh *et al. *[37]. The pH was adjusted to 5 with 0.25 M NaOH, and the Erlenmeyer flask was sealed with a cotton plug and incubated at 30°C for 19-24 h on a rotary shaker.

#### Aerobic cultivation

The aerobic cell cultivation was performed in two steps. The cells were first cultivated in batch mode on a glucose solution, after which the mode was changed to fed-batch with a feed that contained hydrolysate liquid from the pretreatment step. Adapting the yeast cells to pretreatment hydrolysate has previously been shown to make the yeast more resistant to the inhibitors in the fermentation medium and thus give higher ethanol yields in SSF, especially at higher DM contents [38]. Both steps were performed in a 2 L fermentor (Infors AG, Bottmingen, Switzerland) at 30°C. The pH was continuously adjusted to 5 by the addition of 2.5 M NaOH throughout the cell cultivation process.

The working volume for batch cultivation was 500 mL, and the medium contained 20.0 g/L glucose, 22.5 g/L (NH_4_)_2_SO_4_, 10.5 g/L KH_2_PO_4 _and 2.2 g/L MgSO_4 _7H_2_O, 60 g/L trace metal solution and 6.0 g/L vitamin solution. Cultivation was started by adding 60 ml inoculum. Batch cultivation was performed at a stirrer speed of 700 rpm. The fermentor was aerated, and the air flow was adjusted to ensure a concentration of dissolved oxygen of > 5% at all times.

Once the concentration of dissolved oxygen increased rapidly, indicating that all the ethanol produced during batch cultivation had been depleted, batch cultivation was changed to fed-batch cultivation. This occurred 21-22 hours after start of the aerobic batch cultivation in the various cultivation batches. Fed-batch cultivation was performed with hydrolysate from the pretreatment step. A total volume of 1 L feed containing hydrolysate supplemented with glucose and salt solution, to give a feed concentrations of 80 g/L glucose, 11.3 g/L (NH_4_)_2_SO_4_, 5.3 g/L KH_2_PO_4 _and 1.1 g/L MgSO_4 _7H_2_O, was added over a period of 16-24 hours. The final concentration of hydrolysate in the fermentor was equivalent to that which would have been obtained if the slurry from pretreatment had been diluted to 7.5% WIS. Fed-batch cultivation was performed in the aerated fermentor at a stirrer speed of 1000 rpm.

#### Cell harvest

The cultivation medium was centrifuged in 750 mL containers at 3500 rpm for 5 min (Jouan C4-12 centrifuge, St Herblain, France). The time elapsed between cell harvest and the addition of the cells to SSF was < 2 h.

### SSF

All SSF experiments were performed in 2 L fermentors (Infors AG, Bottmingen, Switzerland) for 120 hours, with a working weight of 1.3 kg. The temperature in the reactor was maintained at 37°C, and the pH was continuously adjusted to 5 with 2.5 M NaOH. In the batch experiments, the diluted slurry was autoclaved at 121°C for 20 min. In the fed-batch experiments, the slurry in the fermentor at start-up was autoclaved in the same way, whereas the substrate feed was not autoclaved. The substrate intended for the feed was pressed (Tinkturenpresse HP-5M, Fischer Maschinenfabrik GmbH, Neuss, Germany) to the WIS concentrations given in Table 1. Nutrients were mixed together, sterilized and added to the reactor to give final concentrations of 0.5 g/L (NH_2_)_2_HPO_4_, 0.025 g/L MgSO_4 _7H_2_O and 1.0 g/L yeast extract. The SSF experiments were performed with a yeast cell concentration of 5 g dry yeast cells/kg final working weight. A commercial cellulase mixture was used, consisting of a cellulase derived from *Trichoderma reesei *(Celluclast 1.5L; Novozymes A/S, Bagsværd, Denmark) (57.8 filter-paper units (FPU)/g and 38 IU/g) supplemented with a β-glucosidase preparation (Novozyme 188; Novozymes A/S) (503 β-glucosidase IU/g). The level of enzymes added corresponded to a total cellulase activity of 5 FPU/g WIS and a total β-glucosidase activity of 8 IU/g WIS.

**Table 1:** Summary of the experiments performed

Experiment	WIS, %	Batch of pretreated material	Mode of SSF	Type of slurry in batch	WIS of feed, %
1A	10	1	Batch	Whole, pressed slurry	-
1B	6-10	1	Fed-batch	Whole slurry	29.5
1C	6-10	1	Fed-batch	Whole slurry	29.5^a^
1D	6-10	1	Fed-batch	Whole slurry	29.5^a^
2A	14	2	Batch	Whole, pressed slurry	-
2B	9-14	2	Fed-batch	Whole slurry	24.1
2C	9-14	2	Fed-batch	Whole slurry	24.1^a^
2D	9-14	2	Fed-batch	Whole slurry	24.1^a^
3A	14	2	Batch	Whole, pressed slurry	-
3B	9-14	2	Fed-batch	Whole, pressed slurry	23.6
3C	9-14	2	Fed-batch	Whole, pressed slurry	23.6^a^
3D	9-14	2	Fed-batch	Whole, pressed slurry	23.6^a^
4A	14	2	Batch	Washed, pressed slurry	-
4B	9-14	2	Fed-batch	Washed, pressed slurry	19.9
4C	9-14	2	Fed-batch	Washed, pressed slurry	19.9^a^

^a^Including enzymes.

**Figure 2:**
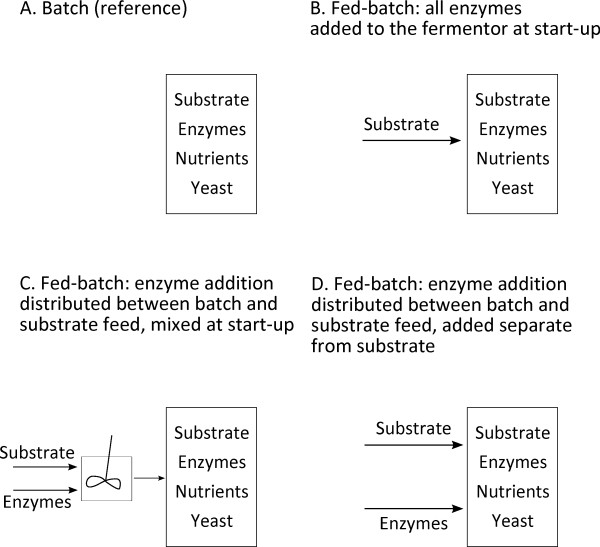
**The four enzyme feeding strategies investigated**.

The following enzyme feeding strategies were investigated.

(A) Batch SSF (reference) (Figure 2A).

(B) Fed-batch SSF with all enzymes added to the fermentor at start-up. To make this comparable with feeding strategies (C) and (D), water equal to the amount of enzyme solution added with the feed in (C) and (D) was mixed with the substrate feed (Figure 2B).

(C) Fed-batch SSF with enzymes divided between the batch and substrate feed according to the WIS content in these. The enzymes were mixed with the substrate feed at start-up (Figure 2C).

(D) As in (C) above, but with the difference that the enzymes were added at the same time as the substrate feed but were not mixed with the substrate before addition to the reactor (Figure 2D).

In fed-batch SSF, the feed was added manually in four equally sized portions at 4, 5.5, 7 and 8.5 hours after start-up, as early addition of substrate has been shown to give better results than late addition [33,39]. Experimental series were run at final WIS concentrations of 10% and 14% (in fed-batch mode starting with 6% and 9% WIS, respectively). Runs with 10% WIS were performed on whole pretreated slurry (SSF 1A to 1D in Table 1). Runs with 14% WIS were performed on whole pretreated slurry (SSF 2A to 2D in Table 1), on slurry with 20% lower inhibitor concentration (obtained by replacing part of the hydrolysate with water) (SSF 3A to 3D in Table 1) and on washed slurry (SSF 4A to 4C in Table 1) (obtained by diluting and washing with excess water several times).

### Analysis

All analyses were performed in duplicate. DM content was determined by drying the samples in an oven at 105°C until a constant weight was obtained. The composition of the spruce and of the washed solids from the pretreated slurry was determined according to the National Renewable Energy Laboratory (NREL) procedure for the determination of structural carbohydrates and lignin in biomass [40]. The solids used for analysis were separated from the liquid fraction of the pretreated slurry by filtration and washed with excess water, making sure that the liquid fraction in the material was replaced several times during the washing process. The hydrolysate from the pretreated slurry was analyzed for its content of oligosaccharides using the NREL procedure for the determination of sugars, byproducts and degradation products in liquid fraction process samples [41]. The oligosaccharide concentration was determined as the difference in monomeric sugar concentration before and after acid hydrolysis.

Samples from analysis of the raw material and the washed solids, the hydrolysate of the pretreated slurry, and samples from SSF were analyzed for their content of monomeric sugars. All samples were filtered through a 0.2 μm filter to remove particles before analysis. Analyses were carried out using a high-performance anion-exchange chromatograph (HPAEC) coupled with pulsed amperometric detection (PAD) and an electrochemical detector (ED40; Dionex, Sunnyvale, CA, USA). A gradient pump (GP40), an autosampler (AS50), a guard column (Carbo Pac PA1) and an analytical column (PA10) (all Dionex) were used. The eluent was 2 mM NaOH at a flow rate of 1 mL/min, and the injection volume was 10 μL.

The samples taken from the SSF experiments were also analyzed for their byproduct content (lactic acid, acetic acid, 5-hydroxymethylfurfural and furfural) and ethanol using high-performance liquid chromatography (HPLC), a refractive index detector (Shimadzu, Kyoto, Japan) and strong cation exchange resin column (Aminex HPX-87H; Bio-Rad Laboratories, Hercules, CA, USA) at 65°C with 5 mM H_2_SO_4 _as eluent at a flow rate of 0.5 mL/min.

## Results

Unless otherwise stated, the ethanol yield is given as the ethanol yield from the SSF step, and is expressed as a percentage of the theoretical yield, based on the contents of glucose and mannose in the pretreated material.

### Pretreatment

The DM content of the wood chips before pretreatment was 48%. After pretreatment, the slurries had a DM content of 13.3% (batch 1) and 16.0% (batch 2) WIS. The compositions of the raw material and the two pretreated batches are presented in Table 2 and Table 3. Most of the differences in concentrations of sugars in the material of the two pretreated batches were due to different correction factors for sugar degradation in the experimental procedure used in the material analysis. This has no influence on the results of the study, as the same material was used within each experimental SSF series.

**Table 2:** Composition of the raw material (spruce) and the washed fibers from the two batches of pretreated slurry

Component	Amount, % of dry matter		
	Raw material	Washed pretreated material	
		Batch 1	Batch 2
Glucan	44.9 ± 0.1	46.7 ± 1.5	53.4 ± 0.4
Mannan	12.0 ± 0.0	1.9 ± 0.3	0.7 ± 0.0
Xylan	5.2 ± 0.0	1.6 ± 0.1	0.4 ± 0.0
Galactan	2.2 ± 0.0	1.2 ± 0.1	0.1 ± 0.0
Arabinan	2.0 ± 0.0	1.2 ± 0.0	0.0 ± 0.0
Lignin	31.1 ± 1.2	44.9 ± 2.6	45.3 ± 0.2

**Table 3:** Composition of the liquid fraction of the two batches of pretreated material

Component	Concentration, g/L	
	Batch 1	Batch 2
Glucose	13.3 ± 0.8 (91.7)^a^	27.1 ± 0.1 (42.1)^a^
Mannose	22.1 ± 2.0 (88.3)^a^	28.7 ± 0.1 (41.7)^a^
Xylose	8.6 ± 0.8 (101.5)^a^	11.4 ± 0.0 (47.5)^a^
Galactose	3.6 ± 0.1 (89.0)^a^	4.5 ± 0.0 (42.0)^a^
Arabinose	ND	ND
HMF	1.93 ± 0.0	2.72 ± 0.0
Furfural	0.85 ± 0.0	1.16 ± 0.0
Lactic acid	3.80 ± 0.0	4.75 ± 0.0
Acetic acid	4.92 ± 0.0	5.87 ± 0.0

HMF = 5-hydroxymethylfurfural; ND = not done.

Data are mean values ± SD unless otherwise indicated.

^a^Mean value ± SD, the percentage of sugars in monomeric form compared with total sugars is given in brackets.

### SSF

#### Final WIS concentration of 10%

**Figure 3:**
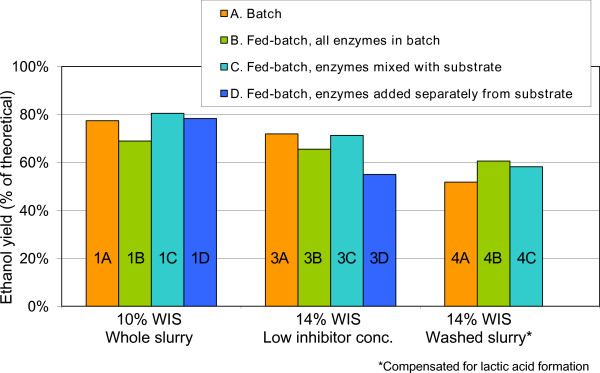
**Ethanol yields (% of theoretical) in SSF with different enzyme feeding strategies**.

Batch SSF with 10% WIS resulted in an ethanol yield of 77.4%. Fed-batch SSF at this fiber concentration with all enzymes added in the batch phase at start-up (Table 1, SSF 1B) resulted in a lower ethanol yield (68.9%). Adding part of the enzyme mix with the substrate feed in fed-batch SSF (Table 1, SSF 1C and 1D) resulted in slightly higher ethanol yields than the batch SSF, both when the enzymes were mixed with the substrate before addition to the fermentor and when they were added separately (Figure 3).

#### Final WIS concentration 14%

##### Whole slurry

Experiments with a final WIS content of 14% using whole pretreated slurry in SSF (Table 1, SSF 2A to 2D) resulted in the accumulation of substantial amounts of glucose in the reactor (around 15 g/L for SSF 2A and 2B and 35 and 45 g/L for SSF 2C and 2D after 120 hours). These results were therefore not used for evaluation of the effect of the enzyme feeding strategy on the ethanol yield in fed-batch SSF.

##### Low inhibitor concentration

SSF at a final WIS content of 14% and low inhibitor concentration resulted in a similar trend to that observed with a final WIS content of 10%, with similar ethanol yields for batch SSF and fed-batch SSF when part of the enzyme mix was added with the substrate feed (mixed) and a slightly lower ethanol yield for fed-batch SSF when all the enzymes were added to the fermentor at start-up (Figure 3). In this case, fed-batch with enzyme feeding strategy 'D' (part of the enzymes added with the substrate feed, but separately from the substrate) resulted in a lower ethanol yield than both the batch and the other fed-batch experiments (Figure 3).

##### Washed slurry

SSF at a final WIS content of 14% and washed slurry resulted in the formation of lactic acid, starting between 10 and 24 hours after start-up in all cases (Table 1, SSF 4A to 4C). The final lactic acid concentration after 120 hours was around 18 g/L in all of these SSF runs. The ethanol yields for these SSF runs have been compensated for lactic acid formation, assuming that the sugars used for the observed lactic acid formation could instead have been converted to ethanol with 100% yield. Batch SSF (Table 1, SSF 4A) resulted in an ethanol yield of 51.8%. Both fed-batch SSF when all enzymes were added at start-up (Table 1, SSF 4B) and when part of the enzyme mix was added with the substrate feed (mixed with the substrate feed) (Table 1, SSF 4C) resulted in higher ethanol yields than batch SSF. The ethanol yield was slightly higher when all enzymes were added in the batch phase at start-up (Figure 3).

#### Initial productivity

**Figure 4:**
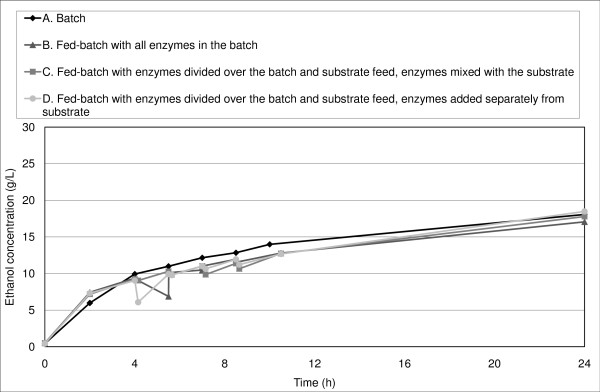
**Ethanol concentration in SSF with a final WIS content of 10% and whole slurry during the first 24 hours**.

**Figure 5:**
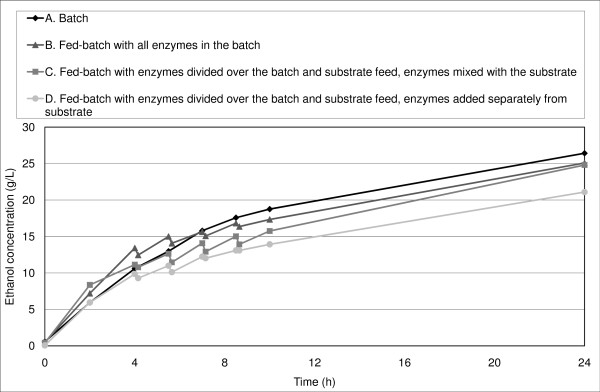
**Ethanol concentration in SSF with a final WIS content of 14% and low inhibitor concentration during the first 24 hours**.

**Figure 6:**
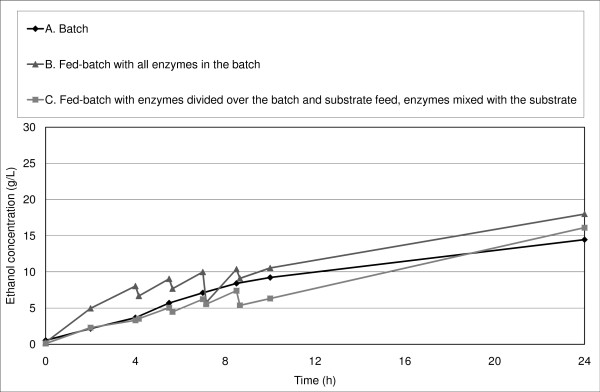
**Ethanol concentration in SSF with a final WIS content of 14% and washed slurry during the first 24 hours (compensated for lactic acid formation)**.

The initial ethanol productivity was equal or slightly higher in fed-batch SSF experiments than in batch SSF (Figure 4, Figure 5, Figure 6).

## Discussion

Running SSF in fed-batch instead of batch mode greatly increased the mixing in the reactor, especially at the higher WIS concentration studied. Furthermore, in all cases, the enzyme feeding strategy had an effect on the ethanol yield in SSF. However, the enzyme feeding strategy resulting in the highest ethanol yield was different not only for the different substrate concentrations studied, but also for different inhibitor concentrations. One explanation of this could be the difference in the degree of deactivation of the enzymes in the different cases. In the experiments with hydrolysate present in the fermentor (Table 1, SSF 1A to 1D and 3A to 3D), the enzymes might be partially deactivated by binding to compounds present in the hydrolysate, resulting in a lower quantity of enzymes being available to the fed substrate, thereby decreasing hydrolysis. In SSF with washed slurry (Table 1, SSF 4A-C), no hydrolysate is present in the fermentor, preventing enzyme deactivation by compounds present in the hydrolysate. Thus, if no deactivation of enzymes due to binding to compounds present in the hydrolysate occurs, the highest ethanol yield is obtained in fed-batch SSF when the greatest possible enzyme quantity is present in the fermentor for the longest possible time (Table 1, SSF 4B).

When adding part of the enzyme mix together with the substrate feed, it appears to be advantageous to mix the enzymes with the substrate before addition to the reactor. This indicates that the hydrolysis of the substrate feed has already started before addition to the fermentor at room temperature, or that some of the enzymes added with the substrate feed adsorb to material already in the reactor, rather than to the fed substrate if they are added separate from the fed substrate.

Previous studies on fed-batch SSF have given different results. In some cases, fed-batch SSF resulted in higher ethanol yields than batch SSF [42,43], whereas other groups found no significant difference in ethanol yield [39,44]. One possible explanation of the differences in the observed ethanol yields could be the different experimental methods used. Not only do raw materials and substrate loadings differ between these studies, but the enzyme feeding strategies are also different. Our results suggest that these differences in experimental procedure, such as enzyme feeding strategy and substrate and inhibitor concentrations, could be the reason for the different ethanol yields obtained in these previous studies.

Rudolf *et al. *[39] reported an increase in initial productivity in fed-batch SSF (all enzymes added in the batch at start-up) compared with batch SSF at a final WIS concentration of 10%, although the overall ethanol yield did not differ significantly. They concluded that the yeast was initially inhibited to a greater degree in batch than in fed-batch SSF; however, the hydrolysis rate was so low that the overall fermentation performance was similar in all experiments. In the present study, a small increase in productivity was observed during the first 4 hours of SSF with a final WIS concentration of 10% (Figure 4). The initial ethanol productivity was also slightly higher for many of our fed-batch experiments with a final WIS concentration of 14% (Figure 5, Figure 6), which confirms the results obtained by Rudolf *et al. *[39]. Another explanation of the increase in initial productivity in fed-batch SSF compared with batch mode could be the improved stirring in fed-batch SSF.

## Conclusions

Running SSF in fed-batch mode does not necessarily give higher ethanol yields than running in batch mode, but when the enzyme-adding method is suitable, similar or slightly higher ethanol yields could be obtained in fed-batch SSF compared with batch mode. The appropriate feeding strategy for the enzymes in fed-batch SSF appears to depend on the conditions during SSF; for example, the WIS and inhibitor concentrations. In the present study, the dependence of ethanol yield on enzyme feeding strategy differed not only with WIS content, but also with inhibitor concentration in experiments with the same WIS content.

## Competing interests

The authors declare that they have no competing interests.

## Authors' contributions

KH planned and carried out the experiments, analyzed the results and wrote the paper. GZ participated in the design of the study, helped analyzing the results and contributed to the draft of the manuscript. MG helped to draft the manuscript. All authors read and approved the final manuscript.
